# Medical Society of the County of Albany—Semi-Monthly Meeting

**Published:** 1871-01

**Authors:** James S. Bailey


BUFFALO

Medical and Surgical Journal.

VOL. X. JANUARY, 1871 No. 6.

### Original Communications.

### ART. I.—*Medical Society of the County of Albany—Semi-Monthly Meeting.* Reported by JAMES S. BAILEY, M. D.

The Society met in the City Buildings, in the Justices' Court-room on Monday, December 12th, at 8 o'clock P. M. Dr. William H. Bailey, President, in the chair.

Dr. E. H. Davis reported a case of "Croupous Diphtheria, relieved by mechanical means."

Hattie M——, aet. 11 years, a robust child, October 18th, was seized with sore throat during a severe epidemic of diphtheria prevailing in the Chemung Valley. On the morning of the 9th, I first saw her and found her with high fever, flushed face, swelling of the throat and parotid glands. The throat internally, over the whole surface, presented a dark-red and highly inflamed appearance, with great swelling of the tonsils and considerable tumefaction of the uvula, and soft palate. On the surface of the tonsils were a few small patches of diphtheritic deposit, which soon disappeared. The falling off of this deciduous membrane exposed ash-colored ulcers, which speedily became deep and large in extent, making the pharynx one ulcerating surface. After about fifteen days, when the upper part of the throat had very much improved, there was an accession of croupous symptoms, with loss of voice and difficulty in inspiration. These symptoms continued to increase in violence until she could scarcely be understood; the act of respiration was most exhausting and oppressive. On the 28th, the twentieth day of the attack, I found her with suffused eyes, livid countenance, cool extremities with impending suffocation.

For several days I had used the whalebone and sponge for applying remedies to the throat and larynx, hoping by these means to arrest the progress of the disease downwards. My former experience in cases of croup—also in acute laryngitis—taught me the necessity of prompt action, and induced me to resort again to the use of the probang for relief. To the end of the whalebone was attached a very small conical-shaped sponge, which was saturated with a solution of silver, (xlv. grs. of the crystal to an ounce of water). The patient was placed in a sitting position, with her hands secured and her head firmly held backward, the instrument was readily passed into the larynx and pushed freely onward until it was believed the sponge had reached the bifurcation of the trachea. On withdrawing it I felt some obstruction, which continued to increase, and at the rima glottidis it was firmly retained. I made several attempts by light traction to disengage it, fearing injury to the structure of the larynx or that I might possibly disengage the sponge from the whalebone. I was, however, compelled to use more force to withdraw it; which, as it was done, produced a marked suction sound, audible in any part of a large room. The sponge was encircled by a false membrane, which became entangled as I withdrew the probang. It exhibited a perfect form or model of the larynx and trachea. It was all intact and unbroken, except the lower margin, which was very soft and gelatinous and had been been separated from a similar exudation still lower down. Upon about the middle of this membrane I observed blood-vessels filled with red blood, ramifying its surface; towards the upper part they were very numerous, coursing in every direction, showing a very high and active organization. The tenacity also increased upwards, when it became very thick, strong and resisting. It measured fully three and one-fourth inches in length. By its removal my patient was greatly relieved, and breathed with comparative ease and comfort. From this time she continued to improve and finally made a complete recovery. She recovered her voice in about four weeks from the removal of the membrane, and the use of her limbs

(which had been partially paralyzed) in about three months from the attack of the disease.

There are two points of interest in this case worthy of note: first, that an instrument of small dimensions may be introduced into the larynx with facility, even when narrowed by the presence of a false membrane; second, that any disease of the larynx or trachea, resulting in such a formation, may be prevented from producing asphyxia by a forcible removal of the membrane, without doing any permanent injury to the vocal organs. Allow me to suggest that a delicate instrument might be constructed so small, when closed, as readily to pass the rima glottidis, to be opened and supported by a delicate spring, covered by sponge or some more appropriate substance, so as to entangle and bring away the false membrane with the instrument, by which death, instead of being the rule, might be rendered the exception in the termination of such cases.

Dr. Sabine remarked that Dr. Thorn, of Troy, had performed a similar operation with the same result.

Dr. Thompson asked Dr. Davis if he was sure he introduced the probang in the trachea.

Dr. Davis answered in the affirmative, and said his reasons for thinking so were that his sponge had penetrated to the bifurcation and caught the membrane and pulled it through itself, like the finger of a glove.

Dr. Thompson mentioned the fact that a committee of surgeons had been appointed in the city of New York to witness Dr. Green perform the operation, but could not do so to the satisfaction of the committee; he, himself, had frequently attempted it, but could not succeed.

Dr. Davis thought the greatest difficulty in doing so was because the sponge used was not small enough.

Dr. C. H. Porter mentioned the case of a lunatic at the Alms-house, who was dying of starvation. A stomach-tube not being accessible, Dr. Stimpson and himself introduced a large-sized catheter through the nares and œsophagus into the stomach, and through it introduced nourishment. The second attempt was not so successful. After the introduction of the catheter, the air perceptibly passed through the instrument during the act of respiration.

In mentioning the case to Dr. Hun, he referred him to a similar case occurring in the hands of a French surgeon, the patient dying quite suddenly. A *post-mortem* revealed the trachea filled with beef tea.

Dr. C. A. Robertson remarked, that when a student in Boston under the tuition of Dr. H. I. Bowditch, a great deal was said upon both sides. Dr. Green instructed Dr. B., who was enabled to pass the instrument through the rima glottidis successfully: he also had, while in Dublin, seen Dr. Stokes perform this operation successfully.

Dr. James S. Bailey then addressed the Society upon one of the secondary symptoms of typhoid fever. He remarked there is one secondary symptom of typhoid fever to which I particularly wish to call the Society's attention this evening. It is one that is seldom witnessed in this climate. It is inflammation and suppuration of the salivary glands. We not only see it as a secondary symptom of true typhoid fever, but also witness it as the signal of any acute disease assuming a typhoid condition. There is no definite period for its attack, but commences at the time when the patient is supposed to be thoroughly convalescent—when the pulse has resumed nearly its normal standard and the patient is beginning to manifest a craving for food. You are then surprised to find an aggravation of symptoms; you ask to see your patient's tongue; he cannot protrude it to your satisfaction; the articulation of the jaw is stiffened, and upon closer scrutiny you find, about the burr of the ear, a tumefaction, which in a few hours wonderfully distorts the features. If not successfully combatted, in thirty-six hours this inflammation of the parotid will go on to suppuration and retard the period of convalesence for weeks, and by its excessive and offensive discharge keep the patient reduced. It is not common for both parotids to be affected at the same time, though I have sometimes seen both parotid and even the sublingual gland inflamed and suppurating at the same time. I have resorted to the various remedies suggested for its relief ineffectually; to stimulating frictions of spirits turpentine, counter-irritants, fly blisters, etc., but still in the majority of cases it would progress to suppuration. I finally resorted to the use of the lancet in the following manner, with success: I introduced the lancet at the angle of the jaw to the bone immediately after I discovered the disposition to inflammation. I usually drew a few drops of blood to a teaspoonful, and sometimes even more. At my next visit, if the progress of the tumefaction was not entirely arrested, I repeated the operation, which was generally sufficient to accomplish the object. I have never failed to prevent suppuration if this treatment was adopted within twelve hours after its commencement; a much longer delay would fail to accomplish the purpose.

My attention was recently called to this subject by a lad ten years of age having these symptoms manifested. It is the only instance I have witnessed in this city. It is very common in the southern States, when typhoid fever prevails alarmingly. It was there I learned to successfully treat this exceedingly annoying symptom. Any acute disease in a warm climate is apt, after running its usual course, to assume a typhoid form, and in this condition we have frequently this secondary symptom. We have it in dysentery, pneumonia, yellow fever, and even bilious remittent fever when neglected and allowed to assume this type.

Dr. Bailey, to illustrate, mentioned a case of yellow fever. The invasive fever lasted an unusual period—six days—with a short remission, and when a protracted secondary fever finally presented the condition alluded to, he resorted to his favorite remedy and found that the hemorrhage from the incision was more than usual, but an application of cob-web was made which arrested it. The patient was left in the hands of an experienced nurse, the doctor feeling quite safe and the bleeding had so effectually relieved the patient that he soon fell into a sound sleep and so did the nurse. Upon his next visit, at daybreak, he was astonished to find his patient bleached almost as white as the sheet, his clothing and bedding saturated with blood, and it had run through the mattrass and pooled upon the floor. The patient, however, greeted him with a smile, and expressed himself as feeling much improved. The case convalesced speedily notwithstanding the accident. This is the only time he had met with so excessive a hemorrhage from this operation. Other physicians noticing the success of his treatment adopted it with the same happy result.

Dr. Robertson, had seen in the army, cases assuming a typhoid condition, but had never witnessed the condition of the salivary glands referred to by Dr. Bailey, and would like to inquire of him if true typhoid fever was as frequent in the South as at the North.

Dr. Bailey said, according to his observations, it was much more frequent there and more malignant in its character. It prevailed very malignantly in Alabama during the years 1852–3–4. Whole families were prostrated with this disease, and in many instances more than half of those attacked proved fatal. It seemed to select the highest and most healthful locations for its attack, and no attributable neglect of cleanliness can be assigned as a cause.

Dr. VanDerveer was highly entertained and pleased with Dr. Bailey's remarks, and had no doubt of the efficacy of his treatment. In the District of Columbia he had witnessed secondary symptoms and had used leeches with benefit in arresting them; had not only seen the salivary glands affected, but also the lymphatic glands of neck, axilla and groins, inflame and progress to suppuration.

Dr. C. A. Robertson reported the following case:—He was called six weeks ago, to see a lady with maggot in the ear. She went to a pic-nic on the Helderbergh's, and while seated in the carriage heard a fly buzzing about her ears. She brushed it away and did not think of the circumstance again until after the lapse of a few days, when she felt some irritation in one ear. She consulted a physician in Schenectady, who found these parasites present; they had penetrated the external auditory canal. She had much pain, and blood oozed from the ear. This strange condition continued. Dr. Robertson was then consulted, and examined with an ear speculum, and removed some with aural forceps; they had penetrated beyond the membrana tympani. The lady seemed cleanly and neat. Dr. R. poured sweet oil into the ear, which was retained for a while; shortly, one came to the surface, apparently searching for breath; this gave relief for ten minutes. He observed more, which were extracted with the forceps. The after treatment consisted in syringing the ear with warm water. The opening in the tympanum closed and her hearing became perfect.

Dr. J. S. Bailey mentioned a case that Dr. P. P. Staats had related to him as occurring in his practice during the summer. Dr. Bailey had, himself, treated several cases while living in Texas. They occurred in filthy negroes, who had laid down under the shade of a tree to enjoy a nap, when the fly had deposited its eggs in the nasal cavity, and these maggots were the result. He had treated them successfully by plugging the posterior and anterior nares with an oiled sponge. These maggots were familiarly known in Texas as the screw-worm, and were even common and destructive to cattle and hogs while roaming at large. They frequently originated from slight wounds which caused the flow of only a drop of blood, when the fly would deposit its eggs, which would soon become screw-worms. Their havoc was quite rapid, and they presented the appearance of the seeds in a sunflower, so closely were they packed while at work. The whole secret of treatment was to exclude the air from them when they soon would perish, causing suppuration of the wound when the insect would not again molest it. It was common with stock raisers, when calomel was not at hand, to stuff the cavity formed by them with dry manure, which had the effect described and relieved the animal. The instinctive nature of the hog caused them to wallow in mud and water for hours, which had a similar effect and caused the parasite to perish.

A recess was taken, and after partaking of refreshments the Society adjourned.

